# GW182-Free microRNA Silencing Complex Controls Post-transcriptional Gene Expression during *Caenorhabditis elegans* Embryogenesis

**DOI:** 10.1371/journal.pgen.1006484

**Published:** 2016-12-09

**Authors:** Guillaume Jannot, Pascale Michaud, Miguel Quévillon Huberdeau, Louis Morel-Berryman, James A. Brackbill, Sandra Piquet, Katherine McJunkin, Kotaro Nakanishi, Martin J. Simard

**Affiliations:** 1 St-Patrick Research Group in Basic Oncology, CHU de Québec-Université Laval Research Centre (Hôtel-Dieu de Québec), Quebec City, Québec, Canada; 2 Laval University Cancer Research Centre, Quebec City, Québec, Canada; 3 Department of Chemistry & Biochemistry, The Ohio State University, Columbus, Ohio, United States of America; 4 Program in Molecular Medicine, RNA Therapeutics Institute, University of Massachusetts Medical School, Worcester, Massachusetts, United States of America; University of Cambridge, UNITED KINGDOM

## Abstract

MicroRNAs and Argonaute form the microRNA induced silencing complex or miRISC that recruits GW182, causing mRNA degradation and/or translational repression. Despite the clear conservation and molecular significance, it is unknown if miRISC-GW182 interaction is essential for gene silencing during animal development. Using *Caenorhabditis elegans* to explore this question, we examined the relationship and effect on gene silencing between the GW182 orthologs, AIN-1 and AIN-2, and the microRNA-specific Argonaute, ALG-1. Homology modeling based on human Argonaute structures indicated that ALG-1 possesses conserved Tryptophan-binding Pockets required for GW182 binding. We show *in vitro* and *in vivo* that their mutations severely altered the association with AIN-1 and AIN-2. ALG-1 tryptophan-binding pockets mutant animals retained microRNA-binding and processing ability, but were deficient in reporter silencing activity. Interestingly, the ALG-1 tryptophan-binding pockets mutant phenocopied the loss of *alg-1* in worms during larval stages, yet was sufficient to rescue embryonic lethality, indicating the dispensability of AINs association with the miRISC at this developmental stage. The dispensability of AINs in miRNA regulation is further demonstrated by the capacity of ALG-1 tryptophan-binding pockets mutant to regulate a target of the embryonic *mir-35* microRNA family. Thus, our results demonstrate that the microRNA pathway can act independently of GW182 proteins during *C*. *elegans* embryogenesis.

## Author Summary

Animal cells possess different small RNA species capable of precisely controlling the gene expression. Among them, microRNAs form a silencing complex with an Argonaute protein (known as miRISC) that abrogates protein production by targeting specific messenger RNAs. While there is a consensus that miRISCs are effective to mediate gene silencing, it is still unclear if they exist in different types in animals. Here we report specific mutations in the *C*. *elegans* microRNA-specific Argonaute ALG-1, which alter its association with the orthologs of GW182 proteins, important factors for miRISC-mediated silencing. Our genetic characterization of this mutant shows that part of miRISCs function without the GW182 orthologs during the embryogenesis. These findings suggest the presence of distinctive miRISC that can regulate gene expression in different ways during animal development.

## Introduction

MiRNAs are highly conserved small non-coding RNAs that orchestrate gene expression in a broad range of developmental processes. The production of miRNA implicates a successive two-step processing involving two RNase III enzymes, Drosha and Dicer, which cleave the primary and precursor miRNA molecules in the nucleus and cytoplasm, respectively. The 21–23 nucleotide RNA products are loaded onto Argonaute proteins to form the ribonucleoprotein complex referred to as microRNA induced silencing complex or miRISC (Reviewed in [1], [2]). Utilizing sequence complementarity, the miRNAs then guide the miRISC to the 3' untranslated region (3'UTR) of target mRNAs to silence their expression. In humans and *Drosophila*, the miRISC is associated with a key partner protein, GW182, which contains glycine-tryptophan (GW) repeats. The GW182 N-terminal domain uses these GW repeats to interact with Argonaute [3–5], while the C-terminal domain recruits the PAN2-PAN3 and CCR4-CAF1-NOT deadenylase complex [6–8]. As a result, the complex triggers mRNA deadenylation and/or translational repression. Despite the difference in their domain organization from that of *Drosophila* and human GW182 proteins, two related *C*. *elegans* proteins, AIN-1 and AIN-2 [9, 10], appear to be orthologs of GW182 in *C*. *elegans* (reviewed in [11]). Both AIN-1 and AIN-2 are known to interact with Argonautes proteins through their GW repeats, but only AIN-1 interacts with PAN and NOT proteins [12] indicating that AIN-1 is most likely the *bona fide* functional GW182 ortholog. Thus, the interaction between Argonaute and GW182 proteins is clearly important for miRNA-mediated gene silencing across species, although the domain architectures of GW182 proteins are varied among species.

Argonaute proteins have a bilobed structure, each composed of the N and PAZ domains or the MID and PIWI ones (Reviewed in [13]). The PAZ and MID domains are engaged in the guide-RNA recognition at the 3’ and 5’ ends, respectively, while the PIWI domain harbors an RNase H-like active site that catalyzes the endonucleolytic cleavage of nucleic acids. Besides conferring the “slicing activity” on some Argonautes, the PIWI domain has also been reported to be important for the recruitment of silencing factors such as GW182 [4, 14, 15]. The crystal structure of human Argonaute2 (hAgo2) identified two hydrophobic pockets on the surface of the PIWI domain that were occupied with free tryptophan residues, suggesting that GW182 proteins could be tethered to hAgo2 via these two pockets on the PIWI domain [16]. The physical interaction between hAgo2 and GW repeats was subsequently validated by NMR studies [17]. Furthermore, analogous binding pockets were also identified on the surface of human Argonaute 1 (hAgo1) [18], suggesting that these pockets could be a conserved feature for recruiting GW-proteins. Notably, although the molecular interactions between Argonaute and GW182 proteins have become clearer in recent years, the functional importance of this interaction in the context of miRISC-mediated silencing during animal development has yet to be determined.

We set out to address the necessity and function of GW182 proteins during miRISC-mediated gene silencing throughout animal development. To achieve these goals, we generated transgenic *C*. *elegans* strains expressing an ALG-1 mutant (ALG-1TPmut) whose Tryptophan-binding Pockets lost an interaction with AIN-1 and AIN-2. We have demonstrated that the tryptophan-binding pockets are required for its interaction with AIN-1 and AIN-2. Surprisingly, loss of the physical interaction between ALG-1 and AINs phenocopied the null allele of *alg-1(0)*, whereas embryonic lethality due to lack of *alg-1* and *alg-2* was rescued by *alg-1(TPmut)* alone. These results indicate the existence of a type of miRISC that plays an essential role, without the aid of GW182 proteins, during embryogenesis in animal development.

## Results

### ALG-1 tryptophan-binding pockets are essential for the interaction with GW182 proteins AIN-1 and AIN-2

**Fig 1:**
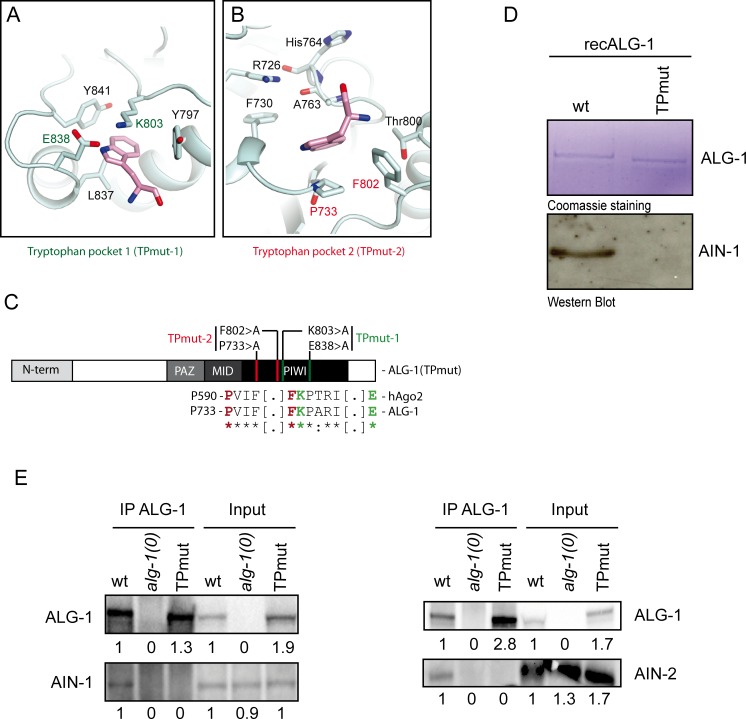
The Tryptophan-binding pockets of ALG-1 are essential to mediate the interaction with AIN-1 and AIN-2 proteins *in vitro* and *in vivo*. **(A and B) Putative Tryptophan-binding pockets of ALG-1.** A homology model (pale cyan) of ALG-1 tryptophan-binding pockets 1 (A) and 2 (B) was generated by Swiss-Model from 4OLB and superposed on the tryptophan (pink)-bound hAgo2 structure (PDB code: 4OLB). hAgo2 is not shown for clarity. **(C) Schematic view of ALG-1 structured protein domains.** The residues forming the tryptophan-binding pocket 1 and 2 are coloured in green and red respectively and mutated into non-polar amino acid alanine to generate an ALG-1 Tryptophan-binding Pockets mutant (hereafter named TPmut). **(D) GST pull-down assays using bacterially expressed GST-Tagged wild-type or tryptophan-binding pocket mutants of ALG-1 protein.** An AIN-1 peptide fragment containing the AGO Binding Domain (350-641aa) was purified and co-immunoprecipitated with recombinant GST-tagged ALG-1 or ALG-1(TPmut) protein. The upper part of gel was stained with Coomassie Brilliant Blue and the lower blotted with antibody against AIN-1. **(E) Co-Immunoprecipitation of ALG-1 wild-type or mutant and AIN-1 and AIN-2 proteins from whole *C*. *elegans* extract.** Transgenic animals were generated using MosSCI single copy insertion of mCherry-tagged ALG-1 wild-type or TP mutant (TPmut). Animals were then crossed into *alg-1(0)* genetic background. ALG-1 proteins from young adults animals was co-immunoprecipitated using ALG-1 specific antibody and AIN-1 and AIN-2 proteins were detected by Western Blotting. Inputs represent the equivalent of 20% and 25% of the total protein extracts used for the IPs for detecting AIN-1 and AIN-2, respectively. Numbers indicate the relative level of proteins in input and Co-IP compared to wt for each strain.

Recent structural studies of hAgo1 and hAgo2 identified two tryptophan-binding pockets on the exterior of the PIWI domain, which were predicted to serve as the binding site of GW182 proteins [16–18]. Our homology model based on the hAgo structures indicated the presence of two tryptophan-binding pockets on ALG-1 (Fig 1A–1C; S1 Fig). These putative pockets consisted of residues K803 and E838 in the first pocket, and P733 and F802 in the second one, which could recognize tryptophan residues of the bound GW182 protein (Fig 1A and 1B). We tested whether mutations of these residues affected the interaction between ALG-1 and the orthologs of GW182 in *C*. *elegans* both *in vitro* and *in vivo*. First, we purified a recombinant AIN-1 fragment (recAIN-1) encompassing the previously mapped binding site of ALG-1 [12] along with glutathione-S-transferase (GST)-tagged full-length wild-type ALG-1 (recALG-1). We also created at GST-tagged ALG-1 in which the four key residues of the two Tryptophan-binding Pockets were mutated to alanine (hereafter recALG-1(TPmut); Fig 1C and S2 Fig). Consistent with our expectations, the interaction between recALG-1(TPmut) and recAIN-1 proteins *in vitro* was less than detectable by western blotting (Fig 1D), strongly suggesting that the tryptophan-binding pockets on ALG-1 are essential for the interaction with AIN-1.

To test whether tryptophan-binding pockets mutation also disrupts the interaction with AIN-1/AIN-2 *in vivo*, we generated an *alg-1(0)* mutant *C*. *elegans* strain expressing a single copy of either *alg-1* wild-type (wt) or *alg-1(TPmut)* gene under the control of endogenous regulatory elements. We then immunoprecipitated ALG-1 from these animals using a specific antibody [19]. While we could recover both wild-type ALG-1 and ALG-1(TPmut) in our immunoprecipitations, we could only observe an association of AIN-1 and AIN-2 with wild-type ALG-1 (Fig 1E and S3 Fig). These results prove that ALG-1 binds to AINs through the tryptophan-binding pockets, in a manner consistent with the interaction between human Argonautes and GW182 [17].

### The interaction between ALG-1 and AIN proteins is not essential for miRISC formation

**Fig 2:**
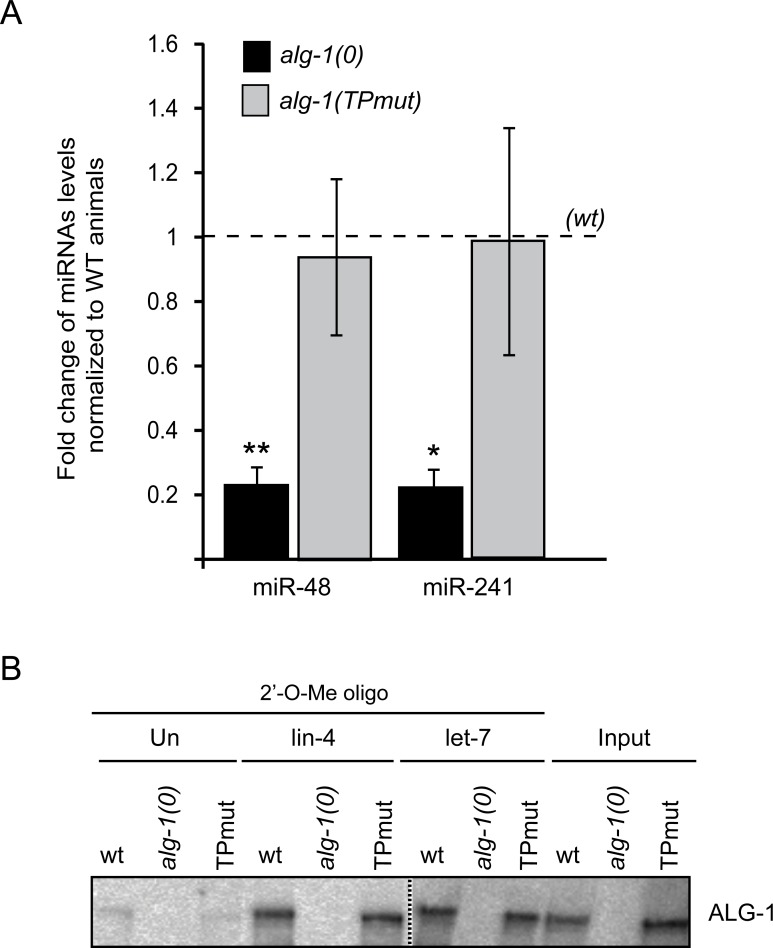
The loss of function of ALG-1 tryptophan-binding pockets mutant occurs downstream of the biogenesis of microRNA. **(A) Quantification of microRNA expression**. The levels of miR-48 and miR-241 were measured by quantitative RT-PCR at mid L3 stage from *alg-1TPmut* and *alg-1(0)* whole worm RNA extracts, and were normalized to *alg-1(wt)*. Small nucleolar RNA sn2841 was used as reference. The error bars represent the 95% confidence interval from three independent experiments and a Student’s two-sided t-test was applied to obtain *p* values. *p<0.05, **p<0.005. **(B) Western Blot analysis of ALG-1(TPmut) or (wt) associated to lin-4 and let-7 miRISC**. Isolation of miRISC was performed using a 2′-O-methyl oligonucleotide (2′-O-Me oligo) column complementary to lin-4 or let-7 mature microRNA. The extract from transgenic animals was first pre-incubated with an unrelated 2′-O-methyl oligonucleotide column (Un) then incubated with the indicated 2′-O-methyl oligonucleotide complementary to lin-4 and let-7 sequences. The purified complexes were run on to an SDS polyacrylamide gel and blotted with an antibody against ALG-1. The dashed line indicates that unrelated lanes have been removed between samples.

It is well known that the alteration of miRNA-specific Argonautes causes significant effects on the levels of miRNAs [20]. In *C*. *elegans*, the loss of *alg-1* gene leads to a dramatic decrease of mature miRNAs along with the accumulation of miRNA precursors, suggesting a role in miRNA processing [21, 22]. To assess whether ALG-1 tryptophan-binding pockets mutant retains its function in miRNA biogenesis, we investigated the levels of precursor and mature miRNAs in worms carrying null alleles of the *alg-1* gene and expressing either wild-type ALG-1 or ALG-1(TPmut). Quantitative real-time PCR and Northern blotting analyses showed that the level of mature miRNA were reverted to that of wild-type by the expression of ALG-1(TPmut) (Fig 2A and S4A Fig). Accordingly, the levels of miRNA precursors also decreased to those of wild type (S4A Fig). These results indicate that the interaction between ALG-1 and AINs is not necessary for miRNA processing.

Next, we assessed whether the mutations of the tryptophan-binding pockets affect binding of ALG-1 to miRNAs. We used 2'-O-methyl RNA affinity columns to purify ALG-1(TPmut) that had been loaded *in vivo* with miRNAs complementary to the affinity matrix [23, 24]. Comparable amounts of wild-type ALG-1 or ALG-1(TPmut) were associated with lin-4 and let-7 miRNAs (Fig 2B). Conversely, we also observed a similar level of miRNAs bound to different ALG-1 immunoprecipitated complexes (S4B Fig), suggesting that GW182 proteins do not affect the interaction between the Argonaute and miRNAs. Thus, these results suggest that GW182 proteins are dispensable for miRISC assembly.

### The alteration of tryptophan-binding pockets leads to defective miRNA-mediated regulation in the somatic tissues

**Fig 3:**
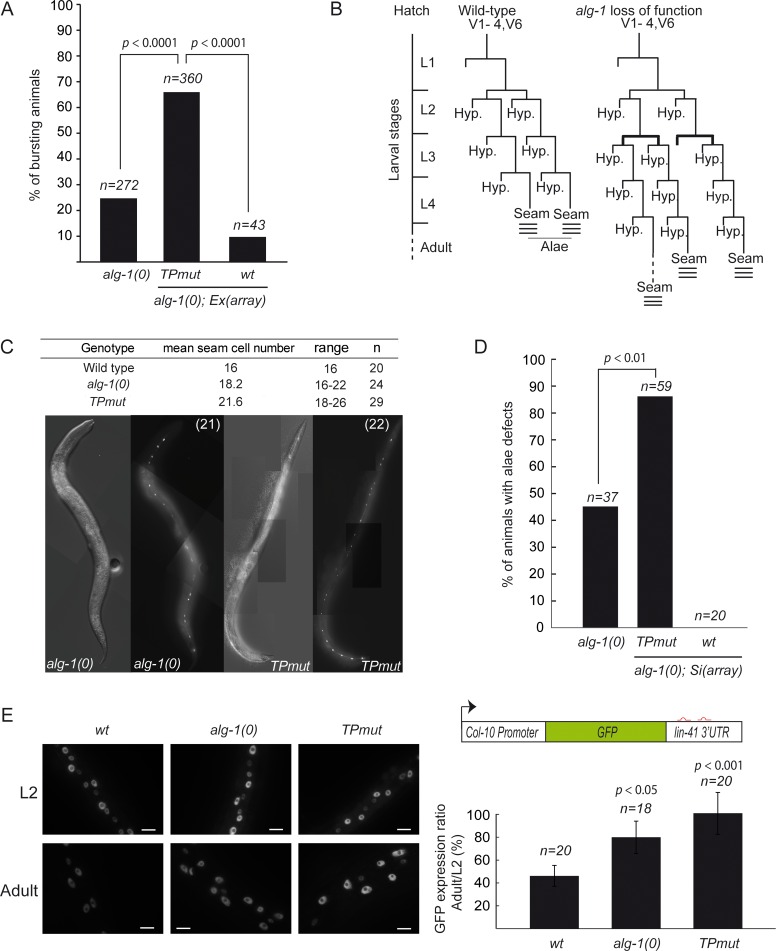
Impairing the interaction with AIN proteins leads to post-embryonic heterochronic phenotypes. **(A) Phenotypic analysis.** The *alg-1* null (*alg-1(0)*) animals were injected with an array (*Ex(array)*)containing plasmids expressing a dominant *rol-6* injection marker (making animals rolling) along with *alg-1(wt)* or *alg-1(TPmut)*,expressing vectors. F1 transgenic rollers animals were picked. The number of bursting animals scored (n) 48h later is indicated. The two-tailed *p* value indicated was measured by Fisher’s exact test. **(B) Seam cells lineage of wild type or *alg-1* mutant animals**. The extra-proliferative division pattern at the L3 division stages of seam cells are shown in bold in *alg-1* mutant. This characteristic of *alg-1* loss of function pattern result in a retarded heterochronic phenotype observable by specific defect such as alae structure synthesis during the L4-adult transition stages (here represented by three lines). **(C) Average and range of seam cells number at adulthood.** The indicated strains were crossed with a strain expressing GFP in the seam cells (*scm*::*GFP*) and the number of seam cells scored in young adult. The mean value and range covered by the individual counts are indicated. The number of animal scored (n) is indicated. **(D) Analysis of animals alae.** The structure of alae of young adult *alg-1(0)* animals expressing either *alg-1wt* or *alg-1(TPmut)* single inserted (Si) transgenic allele were assayed by Normarski DIC imaging and compared to *alg-1(0)*. The number of animal scored (n) is indicated. The two-tailed *p* values indicated were measured by Fisher’s exact test. **(E) Functional analysis using a let-7 microRNA reporter**. Animals carrying the *GFP* reporter transgene under the control of a hypodermis-specific *col-10* promoter and the *lin-41* 3’UTR containing the *let-7* miRNA binding sites (red; diagram) were crossed into either *alg-1(0)* or *alg-1(TPmut)* animals. **Left:** Representative pictures of GFP expression during early larvae stages (L2) and in young adult animals (Adult). Scale bars represent 10μm. **Right:** Quantification of GFP in adult relative to L2 stage animals. The quantification of the GFP signal was performed by measuring the mean of the GFP detected in five different cells for each animal. The number of animals scored (n) is indicated. The error bars represent the 95% confidence interval from three independent experiments and a Student’s two-sided t-test was applied to obtain *p* values.

We next investigated whether AINs-free miRISC can still control gene expression in animals. To this end, we took advantage of developmental phenotypes caused by loss of specific miRNAs. It is known that loss of let-7 miRNA family in *C*. *elegans* causes a characteristic phenotype, in which the animal bursts from the vulval opening after L4 moult [25]. This lethal phenotype was observed in a fraction of the population of worms deficient for *alg-1* (Fig 3A). The addition of extra chromosomal transgene arrays expressing wild-type *alg-1* gene significantly reduced the number of animals that burst (Fig 3A). In contrast, the expression of ALG-1(TPmut) did not rescue *alg-1(0)* animals (Fig 3A). In both the strains, the expression levels of AIN proteins were comparable (S5 Fig).

To further characterize ALG-1(TPmut) in miRNA-mediated gene silencing, we monitored the developmental patterning of seam cells. These lateral rows of hypodermal cells undergo a postembryonic developmental program, consisting of patterned rounds of division during each larval stage (L1 to L4), and ended by terminal differentiation encompassing exit from the cell cycle, cell fusion and production of a cuticular structure (called alae) at the adult developmental transition (Fig 3B). This developmental program is controlled at different larval stages by lin-4 miRNA [26], the let-7 family miRNAs (miR-48, miR-84, miR-241 and let-7) [25, 27] and their targets *lin-14*, *lin-28*, *hbl-1*, *daf-12* and *lin-41* [25, 27–33]. In absence of *alg-1*, the symmetric seam cell division program that occurs once at the L2 stage is repeated, leading to an increase of seam cell numbers and structural defects in cuticular alae caused by inappropriate terminal differentiation (Fig 3B–3D; [21]). Consistent with the lethality caused by the loss of let-7 family regulation, the seam cell developmental phenotype in *alg-1(0)* mutant animals was rescued by the expression of wild-type ALG-1 but not of ALG-1(TPmut) (Fig 3D). Using transgenic animals expressing a GFP reporter under the control of the *lin-41* 3'UTR, a known target of let-7 miRNA regulated at the L4-Adult transition [27, 32], we observed that the repression of *lin-41* by let-7 miRNA is altered in the ALG-1(TPmut)-expressing adult animals (Fig 3E). Interestingly, in all cases the phenotypes observed in ALG-1(TPmut) animals are more severe than the ones caused by a complete loss of ALG-1 proteins. These observations suggest that with its retained capacity of interacting with microRNAs, ALG-1(TPmut) sequester microRNA from ALG-2, the other functional microRNA-specific Argonaute in worms [19, 21].

**Fig 4:**
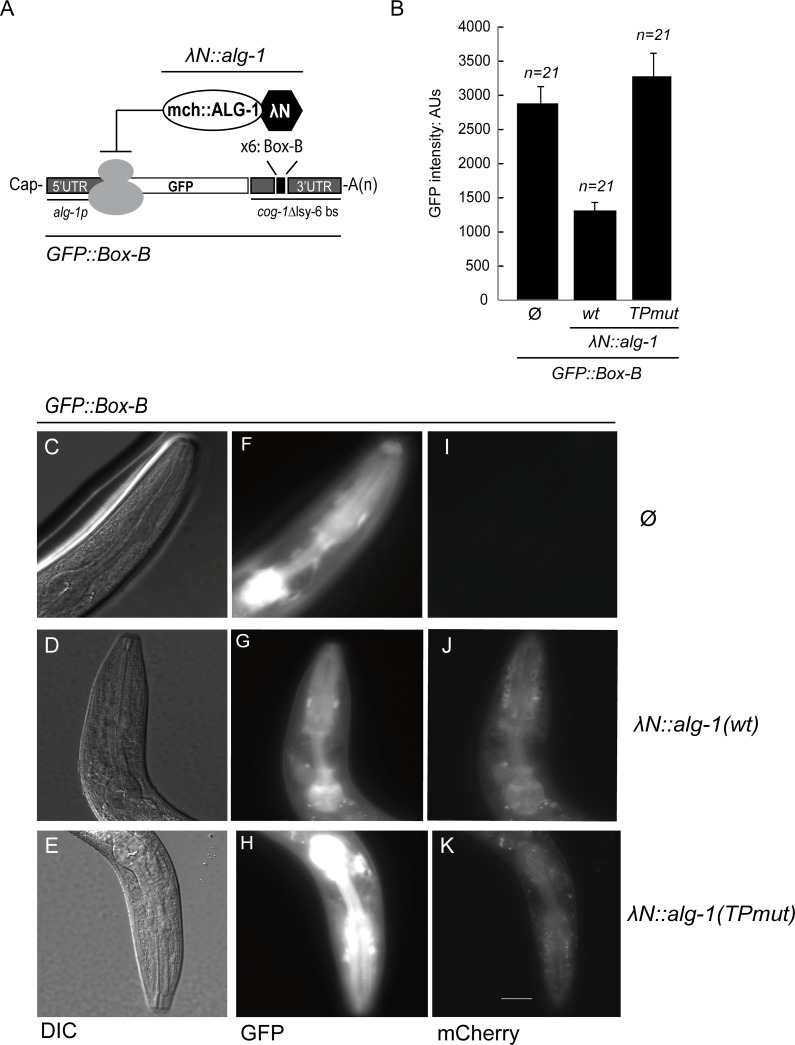
Tethered ALG-1 tryptophan-binding pockets mutant cannot repress gene expression. **(A) Schematic view of the *alg-1p*::*gfp* reporter**. A GFP reporter under the control of the *alg-1* promoter (*alg-1*p) fused with a modified *cog-1* 3’UTR where the *lsy-6* binding sites were replaced by 6 copies of the Box-B elements is shown. The high affinity between the Box-B RNA secondary structure and the λN peptide fused to ALG-1 leads to its recruitment on the reporter in a microRNA independent manner. **(B) Functional analysis of artificial tethering of ALG-1 or ALG-1 (TPmut) to the GFP reporter system in animals**. Using MosSCI, we constructed two transgenic animals carrying two single copy arrays under the same promoter of *alg-1* expressing: 1) GFP fused to the 3'UTR of *cog-1* where the *lsy-6* microRNA binding site was replaced by 6 copies of RNA Box-B element and; 2) a mCherry-tagged ALG-1 (wt) or ALG-1 (TPmut) protein fused to the λN peptide. The strain expressing only the GFP reporter is also shown (∅). The level of GFP expressed in the pharynx was quantified using Arbitrary Units (AU). Images were obtained at the same time of exposure, on the same slide, and with the same area of measure for each transgenic worm. The error bars represent the 95% confidence interval. **(C-K) Representative images of the pharyngeal tethering assays reporter regulation. (F-H)** The expression level of GFP reporter fused to Box-B element from transgenic young adult animals expressing either λN peptide-tagged ALG-1 (G) or ALG-1TPmut (H) was observed into the pharynx at 400X magnification. Transgenic animals expressing only the GFP reporter (∅) are represented in (F). Normarski DIC images are shown in (C-D-E) and expression of mCherry-tagged ALG-1 in respective strains are shown in (I-J-K). Scale bar represents 10 μm. The acquisition time per image is 400ms and 1500ms (GFP and mCherry, respectively).

To assess whether the deficiency of miRNA-mediated gene repression in the ALG-1(TPmut)-expressing animals might be resulting from a defect in binding of target mRNAs, we generated a LambdaN (λN)/Box-B tethering-based reporter that enables interaction of Argonaute with a target, independently of a miRNA-mRNA interaction. In cultured cells, this has been a conventional system to decipher the molecular basis for gene silencing by Argonaute and GW182 proteins [14, 34, 35]. To apply the system to animals, we made a GFP gene reporter where the well-characterized lsy-6 miRNA binding sites in the *cog-1* 3'UTR were replaced by six Box-B stem loop structures (Fig 4A). We then made a transgenic *C*. *elegans* strain co-expressing a single copy of this reporter along with either wild-type or TPmut λN::mCherry-tagged *alg-1* gene, both of which were under the control of the *alg-1* promoter and 3′UTR regulatory regions. We first confirmed that the presence of the N-terminal tag does not affect ALG-1 function by performing *alg-1* mutant rescue. When wild-type λN::mCherry::ALG-1 was co-expressed with the Box-B reporter, a significant decrease of the GFP signal was measured in the pharynx of young adult animals (Fig 4B to 4G). The expression of λN::mCherry::ALG-1(TPmut) protein, however, failed to repress the GFP reporter, suggesting that the interaction with AIN-1 is essential to trigger the repression of the tethering reporter (Fig 4B, 4H). Taken all together, we conclude that the physical contact of ALG-1 to AINs through its tryptophan-binding pockets is important for miRNA-mediated gene silencing in animals.

### The embryos miRISC does not require GW182 proteins to control gene expression

**Fig 5:**
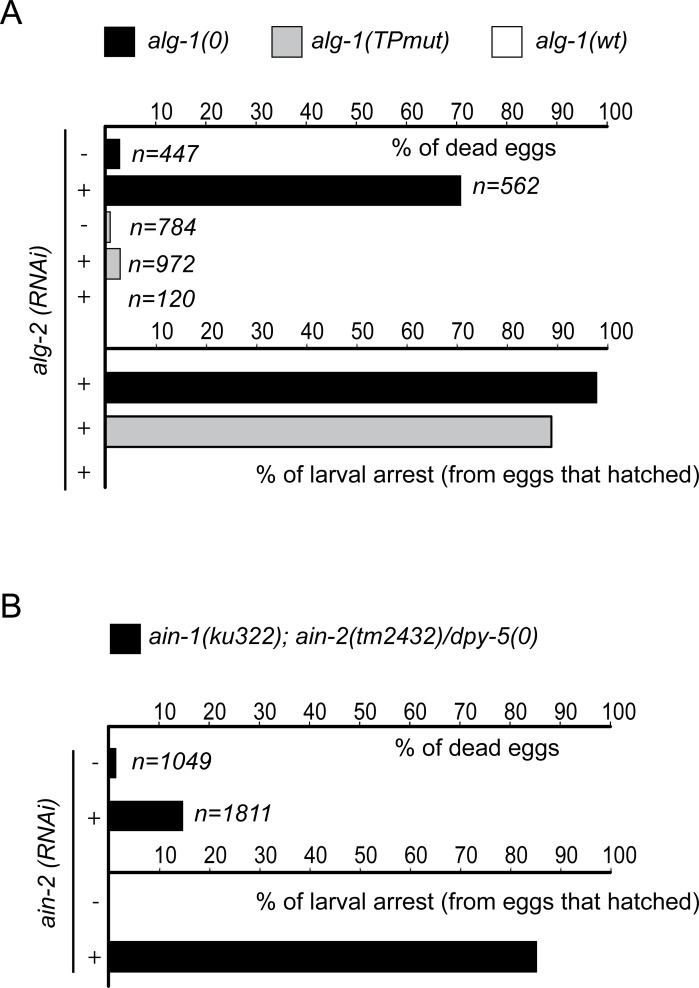
The GW182 proteins are not essential for microRNA-dependent silencing pathway during animal embryogenesis. **(A) The *alg-1* tryptophan-binding pocket mutant is not synthetic lethal with *alg-2***. Early L4 staged *alg-1(0)* animals alone or expressing either *alg-1wt* or *alg-1(TPmut)* transgenic allele were picked on *alg-2 (+)* or *control (empty L4440 vector; -)* RNAi feeding plate for 24h before being separated onto new RNAi feeding plates. F1 progeny animals were scored for embryonic lethality and post developmental defects. The number of F1 animals scored (n) is indicated. **(B) *ain-1* and *ain-2* are not essential for *C*. *elegans* embryonic development.** Early L4 staged *ain-1(ku322); ain-2(tm2432)/dpy-5* animals were picked on *ain-2 (+)* or *control (empty L4440 vector; -)* RNAi feeding plate for 24h before being separated onto new RNAi feeding plates. F1 progeny animals were scored for embryonic lethality and post developmental defects. The number of F1 animals scored (n) is indicated.

The miRNA pathway is a regulatory mechanism that is essential for the control of various steps during animal development including embryogenesis (for reviews see [36–38]). In *C*. *elegans*, this phenomenon is exemplified by the fact that loss of both miRNA-specific Argonaute genes *alg-1* and *alg-2* leads to embryonic lethality [19, 21]. We therefore decided to use our ALG-1 (TPmut) to test whether the interaction between GW182 proteins and miRISC is essential during embryogenesis. To achieve this, we knocked down *alg-2* in *alg-1(0) or (TPmut)* animals by feeding them with bacteria expressing dsRNA against *alg-2*. Consistent with the phenotype observed in simultaneous RNAi knockdown of *alg-1* and *alg-2* [21, 22], we observed that nearly 70% of the F1 *alg-1(0)* population exposed to *alg-2 (RNAi)* displayed embryonic lethality, while the remaining F1 animals arrested just after hatching (Fig 5A). Surprisingly, the expression of ALG-1(TPmut) in an *alg-1(0); alg-2(RNAi)* background is sufficient to rescue embryonic lethality at a level comparable to that of *alg-1; alg-2* loss of function animals expressing a wild-type ALG-1 transgene (Fig 5A). Strikingly, nearly all F1 progeny arrest in early stages of larval development (Fig 5A) suggesting that GW182 proteins are required for miRISC function during larval development but not during embryogenesis.

To directly test the contribution of GW182 proteins for miRNA-mediated silencing during *C*. *elegans* embryogenesis, we constructed a balanced strain with loss-of-function alleles of both AIN genes, (*ain-1(ku322)*; *ain-2(tm2432)/dpy-5)*, exposed them to dsRNA-expressing bacteria targeting *ain-2* (*ain-2 (RNAi)*) to remove all maternally loaded *ain-2* mRNA, and scored the F1 progeny. Even though AINs were well expressed during embryogenesis (S3 Fig and S6 Fig), the alteration of both AIN genes did not cause significant embryonic lethality but rather led to severe larval developmental arrest of the F1 population as seen for *alg-1(TPmut); alg-2* mutant animals (Fig 5B) demonstrating that the alteration of GW182 function in *C*. *elegans* embryos phenocopies the loss of interaction with the miRISC.

**Fig 6:**
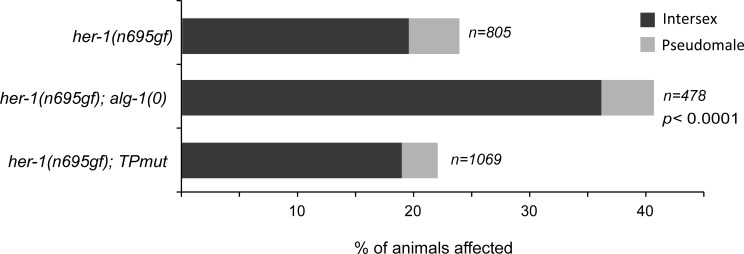
The embryonic sex determination controlled by the *mir-35* microRNA family does not require an AIN-containing miRISC. The fraction of animals displaying pseudomale and intersex phenotypes in the *her-1(n695gf)*, *her-1(n695gf); alg-1(0)* and *her-1(n695gf); alg-1(TPmut)* population were scored. The number of animals scored (n) for each genetic background is indicated. The two-tailed *p* values indicated were measured by Fisher’s exact test.

To further test the importance of AINs in the embryonic miRISC, we tested the capacity of ALG-1(TPmut) to control animal sex determination, an embryonic gene regulatory pathway controlled by the *mir-35-41* microRNA family cluster [39]. We therefore utilized the *her-1(n695gf)* allele that causes a weak derepression of *her-1* expression in *C*. *elegans* hermaphrodites leading to mild masculinization (with a low penetrance of intersex and pseudomale; Fig 6). While the loss of *alg-1* in this sensitized background significantly increased the number of masculinized animals observed, the expression of ALG-1(TPmut) in *her-1(gf)/alg-1(0)* animals completely reestablished it to the level observed in *her-1(gf)* animals (Fig 6) demonstrating that the interaction with AINs is not required for the function of the *mir-35* microRNA family in this embryonic decision. Taken all together, our findings support that the embryonic miRISC does not necessitate GW182 proteins to silence gene expression.

## Discussion

Most of the reported mutations in Argonaute proteins affect the binding of both miRNA and GW182 proteins [3, 40, 41]. Until recently, there have been only two Argonaute mutants reported deficient in the interaction with GW182 protein without affecting miRNA binding [3, 40]. Interestingly, based on our prediction, two point mutations reported in *Drosophila* AGO1 (R771 and F777) could also be involved in forming the same tryptophan-binding pockets 1 and 2 in *C*. *elegans*. In this study, we have generated such point mutations in ALG-1 and demonstrated the functional importance of these pockets to sustain the interaction even with a different type of GW182 proteins that possess a non-canonical domain architecture. Interestingly, a very recent study using culture cell systems reported that the mutation of these binding pockets in *Drosophila* AGO1 and human Ago2 abolished the interaction with their GW182 proteins without affecting microRNA binding [42]. These Argonaute variants have been and can continue to be useful to study the mechanism of miRNA-mediated gene silencing independent of GW182 proteins. Thus, application of Argonaute tryptophan-binding pockets mutant variants will provide novel strategies to uncover new types of gene regulation in animals.

GW182 proteins have been long thought to be essential for miRNA-mediated gene silencing in animals. Recent observations using *Drosophila* S2 cells as well as cell-free systems, however, suggest that GW182 is not always necessary for miRISC-mediated gene silencing. For example, *Drosophila* Ago1 and Ago2, the latter of which mainly associates with an siRNA-duplex, were both able to repress translational reporters in GW182-dependent and–independent manners [43]. Using miRNA-mediated reporter assays with or without polyA tails, Fukaya and Tomari demonstrated that *Drosophila* Ago1 could block translation independently of GW182 [44]. More recently, the Carthew’s group reported that in fly, miRISC retains the silencing activity under conditions lacking GW182 protein (i.e. when nutrients are removed from S2 cell cultured media)[45]. In these experimental conditions, the absence of GW182 still leads to gene silencing that results from the inhibition of either early translation or elongation. These data implied a possible GW182-independent miRNA repression in cell culture though it remained unclear whether this was the case in animal development.

Using *C*. *elegans* as a model, we show here that there are two miRNA-mediated gene-silencing pathways that appear to be necessary for specific time windows during development. Our *in vivo* approach demonstrates that the abrogation of miRISC interaction with GW182 proteins does not cause embryonic lethality as seen in animals lacking miRNA-specific Argonautes, ALG-1 and ALG-2. These data are in striking contrast with the severe developmental phenotypes observed in those animals after hatching. Given that GW182 proteins are not essential for miRISC-mediated regulation during animal embryogenesis, the miRNA-mediated gene silencing may preferentially block translation, instead of deadenylation and mRNA degradation that requires the recruitment of GW182 proteins to the target mRNAs. This model is reminiscent of the observation that gene silencing can occur independently of mRNA deadenylation during zebrafish embryogenesis [46]. Since GW182 proteins are essential and sufficient for mRNA deadenylation and translational repression, the silencing complex without GW182 must limit the turnover rate of the bound mRNAs during embryogenesis. In this case, the dwell time of miRISC to discriminate the proper target mRNAs would be extended, and could be tested by single molecule studies.

Our study discovered that ALG-1 functions, without the aid of AIN-1 and AIN-2, as an essential factor in early stages of development in *C*. *elegans*. This sheds light on the enigmatic miRNA-mediated gene silencing during embryogenesis in animals, which bypasses a regular gene silence pathway that requires GW182 proteins. However, we cannot exclude a possibility that such a GW182 protein-free miRISC plays another unidentified, but critical role during embryogenesis, in addition to gene silencing. We believe that this study lays a strong foundation and experimental context for future studies to understand how and why miRNA-mediated gene silencing pathways are varied at different developmental stages, and how each pathway involves GW182 proteins.

## Materials and Methods

### Generation of transgenic animals and strain maintenance

All *C*. *elegans* strains were cultured and handled using standard methods. The transgenic strains were generated by MosSCI single insertion method [47, 48] or by extrachromosomal non-integrated transgene expression [49]. The strategies to build plasmids as well as strains for this study are listed in S1 Text.

### Expression, purification and pulldown assays of GST fusion proteins

*E*.*coli* BL21 codon+ cells transformed with pGEX plasmids encoding a GST-fusion construct were grown at 37°C. After adding 0.1mM Isopropyl β-D-1-thiogalactopyranoside (IPTG) at OD_600_ = 0.8, the *E*. *coli* cells were grown at 15°C for 16h. Harvested cells were resuspended in STE buffer (10mM Tris pH8, 150mM NaCl, 1mM EDTA, 5mM DTT, 1mM PMSF) supplemented with 2.5% (w/v) of N-Lauryl-Sarkosyl and lysed at 27kPSi in a constant cell disruptor (One Shot Cell Disruptor, Constant System). 1.5% (v/v) final Triton X-100 was added into the cell lysis. GST-tagged ALG-1 proteins were purified under non-denaturing conditions by affinity chromatography using Glutathione Sefinose matrix and quantified on a 8% SDS-PAGE gel.

The pulldown assay was carried out by mixing 100ng of GST::ALG-1 resin coupled protein with 500ng of AIN-1 fragment in binding buffer (100mM Potassium Acetate, 30mM Hepes-KOH pH7, 2mM Magnesium Acetate, 1.5% Triton X-100, 1mM DTT, 1 tablet/10mL Complete Mini Protease Inhibitor without EDTA (Roche)). The mix was incubated for 1h at 4°C with gentle rotation and washed 2 times with high salt PBS buffer (300mM then 500mM NaCl) followed by a non-stringent final wash with PBS only. Beads were resuspended into 2X denaturing Laemmli buffer and loaded on a 4–15% SDS PAGE gel. The upper part containing GST-tagged ALG-1 was stained with Coomassie Brilliant Blue whereas the lower part was immunoblotted with a primary rabbit polyclonal AIN-1 antibody (dilution 1:1000).

### Preparation of protein extracts, immunoprecipitation, miRISC pulldown assay and western blotting analysis

Staged young adults worms were obtained by Alkaline Hypochlorite Solution treatment and plated onto NGM Agar plates seeded with OP50 bacteria. After 4 days at 15°C, animals were harvested in M9 solution and lysed by sonication into ice-cold lysis buffer (100mM KAc, 30mM Hepes-KOH pH7, 2mM Magnesium Acetate, 1mM DTT, 1.5% triton X-100, 1 tablet /10mL Complete Mini Protease Inhibitor without EDTA (Roche)). Immunoprecipitation and miRISC pull-down assays were carried out as described in [50, 51], respectively. The 2′-O-methyl oligonucleotides sequences have been previously described in [23]. Primary rabbit polyclonal ALG-1 and AIN-1 antibodies were used at 1:1000 dilution in PBST supplemented with 5% of milk with overnight incubation at 4°C. AIN-1 and AIN-2 antibodies were generated by injection of two rabbits with either AIN-1 peptide (EQRAPASTEDYHYS) or AIN-2 peptide (GPPDHYYDYSFLG) and affinity purified using the same epitope (Feldan).

### MicroRNAs analysis

RNA preparation and microRNA quantification by quantitative RT-PCR were performed as described in [50]. To quantify the level of microRNA bound to ALG-1, 4mg of total protein extract was used to immunoprecipitate ALG-1. A fraction of 10% was mixed to Laemmli denaturing buffer and loaded on 8% SDS-PAGE. 90% of the remaining beads were treated with 20μg of proteinase K and RNA was extracted using TriReagent (Sigma). Samples were spiked and normalized with 50fmol of human synthetic miR-20a as technical control.

### RNAi experiment

The RNAi of *alg-2* and *ain-2* were carried out by feeding using cDNA fragment cloned into RNAi feeding vector L4440 and expressed into inducible IPTG HT115 (DE3) bacterial strain as described in [52]. The oligonucleotides used to generate the plasmids as well as the different plasmids are listed in S1 Table and S2 Table, respectively.

### Microscopy

DIC Nomarski images and GFP, mCherry fluorescence expressions were collected in animals using a Zeiss AxioCam HRm digital camera mounted on a Zeiss Axio Imager M1 microscope using the same settings for each animal. Intensity of fluorescence in pharynx was measured with Axiovision 4.6 software.

## Supporting Information

S1 Fig Superposition of the homology models of ALG-1 and ALG-2 on the crystal structure of hAGO2. Homology models of ALG-1 (blue) and ALG-2 (pink) were generated by Swiss-Model based on the crystal structure of tryptophan-bound hAGO2-RISC (4OLB). The bound tryptophans are depicted as stick models (red).
(TIF)

S2 Fig Bacterially expressed proteins purification. **(A)** Analyses of AIN-1 (350–641) using size exclusion chromatography **(Top)** and the SDS-PAGE **(Bottom)**. **(B)** Complementary DNA (cDNA) of *alg-1* was fused to glutathione S-transferase (GST) into a pGEX expression vector and bacterially expressed after induction by IPTG. ALG-1(TPmut) was obtained by directed mutagenesis using pGEX::*alg-1*(*wt*) as template. The cytosolic extract of *E. coli* strain expressing GST-Tagged ALG-1(WT) or ALG-1(TPmut) was loaded onto GST-sefinose resin and analyzed by SDS-PAGE. Increasing amount of commercial recombinant Bovine Serum Albumin (BSA) was loaded in parallel.
(TIF)

S3 Fig The Tryptophan-binding pockets of ALG-1 are essential to mediate the interaction with AIN-1 and AIN-2 proteins in the embryos. ALG-1 proteins from embryos were co-immunoprecipitated using ALG-1 specific antibody and AIN-1 and AIN-2 were detected by Western Blotting. Inputs represent the equivalent of 20% of the total protein extracts used for the IPs. The relative enrichment of ALG-1 (wt and TPmut) as well as AIN-1 and AIN-2 in the IPs normalized to respective input signals are indicated.
(TIF)

S4 Fig Comparative analysis of microRNA levels. **(A)** Northern blot detection of let-7 miRNA in wild-type (wt), *alg-1(0)* and *alg-1(0)* animals expressing ALG-1(TPmut). Upon total RNA extraction, let-7 precursor (pre-let-7) and mature miRNA (let-7) forms were detected using probes complementary to mature miRNAs. The tRNA Glycine (tRNA) was probed and used as loading control. **(B)** ALG-1 immunoprecipitations from young adult animals populations expressing either ALG-1(WT) or ALG-1(TPmut) were performed using specific ALG-1 polyclonal antibody. 90% of the isolated complex was used from RNA extraction and quantitative RT-PCR analysis (**Top**) whereas the 10% remaining was analyzed by immunoblotting against ALG-1 (**Bottom**). For controls, immunoprecipitations were also performed with *alg-1(0)* animals. Inputs represent 5% of total protein extracts used for immunoprecipitation. The dashed line indicates that unrelated lanes have been removed between samples. RNA bounds immunoprecipitated complexes were extracted and the level of let-7 and miR-48 was quantified by quantitative RT-PCR. Before extraction, samples were spiked with human miR-20a microRNA and used as a technical control. Data was normalized to ALG-1(WT). The error bars represent the 95% confidence interval from two independent experiments and a Student’s two-sided t-test was applied on the normalized Ct values to obtained *p* values. NS = no significance.
(TIF)

S5 Fig Protein levels of the GW182 protein AIN-1 and AIN-2 from whole worm extracts. Animals population expressing either ALG-1(TPmut) or ALG-1(WT) along with *alg-1* null allele *alg-1(0)* were boiled into SDS loading buffer and loaded onto SDS-PAGE gel for analysis of endogenous AIN-1 (left) and AIN-2 (right) protein levels. Actin was used as a loading control.
(TIF)

S6 Fig Representative expression patterns of AIN-1 and AIN-2 at different developmental embryonic stages. **(B-D-F-H)** Embryos from transgenic worms expressing GFP-tagged AIN-1 or AIN-2 were observed under fluorescent microscope at 400X magnification with 1000ms of exposure (with the exception of panel B: 2000ms) and with Nomarski optics **(A-C-E-G)**. Scale bar: 20 μm.
(TIF)

S1 Table List of oligonucleotides primers used in this study. (DOCX)

S2 Table List of plasmids used in this study. (DOCX)

S1 Text Description of *C*. *elegans* strains as well as supplemental materials and methods. (DOCX)
