# Correction: GW182-Free microRNA Silencing Complex Controls Post-transcriptional Gene Expression during *Caenorhabditis elegans* Embryogenesis

**DOI:** 10.1371/journal.pgen.1006534

**Published:** 2016-12-29

**Authors:** Guillaume Jannot, Pascale Michaud, Miguel Quévillon Huberdeau, Louis Morel-Berryman, James A. Brackbill, Sandra Piquet, Katherine McJunkin, Kotaro Nakanishi, Martin J. Simard

There is an error in the current address for the first author, Guillaume Jannot. ‘UMR 7126’ should read ‘UMR 7216’. The correct current address is: Epigenetics and Cell Fate, Centre National de la Recherche Scientifique (UMR 7216), Université Paris Diderot (Sorbonne Paris Cité), Paris, France.
