# Supplementary figures and images for: GW182-Free microRNA Silencing Complex Controls Post-transcriptional Gene Expression during Caenorhabditis elegans Embryogenesis
Source: PLoS Genet. 2016 Dec 9;12(12):e1006484. doi: 10.1371/journal.pgen.1006484 (PMC5147811; doi:10.1371/journal.pgen.1006484)

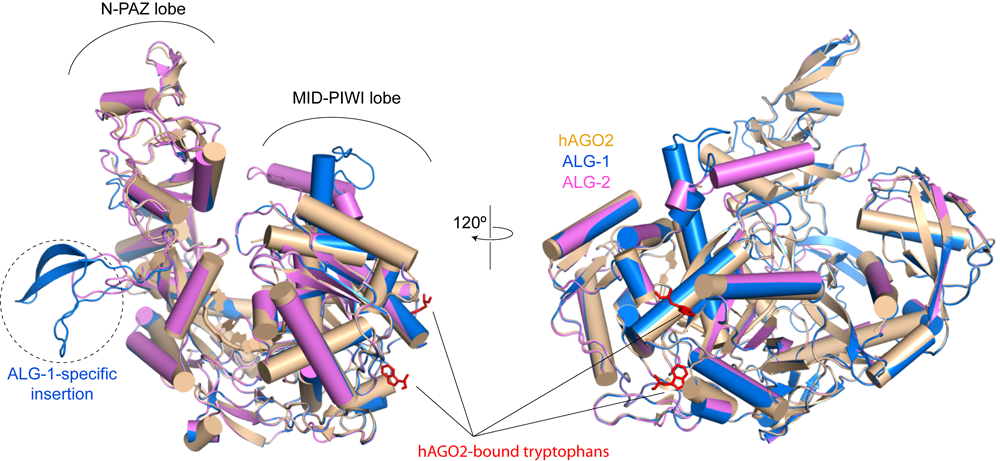

Supplement: S1 Fig — Homology models of ALG-1 (blue) and ALG-2 (pink) were generated by Swiss-Model based on the crystal structure of tryptophan-bound hAGO2-RISC (4OLB). The bound tryptophans are depicted as stick models (red). (TIF) [file pgen.1006484.s001.tif]

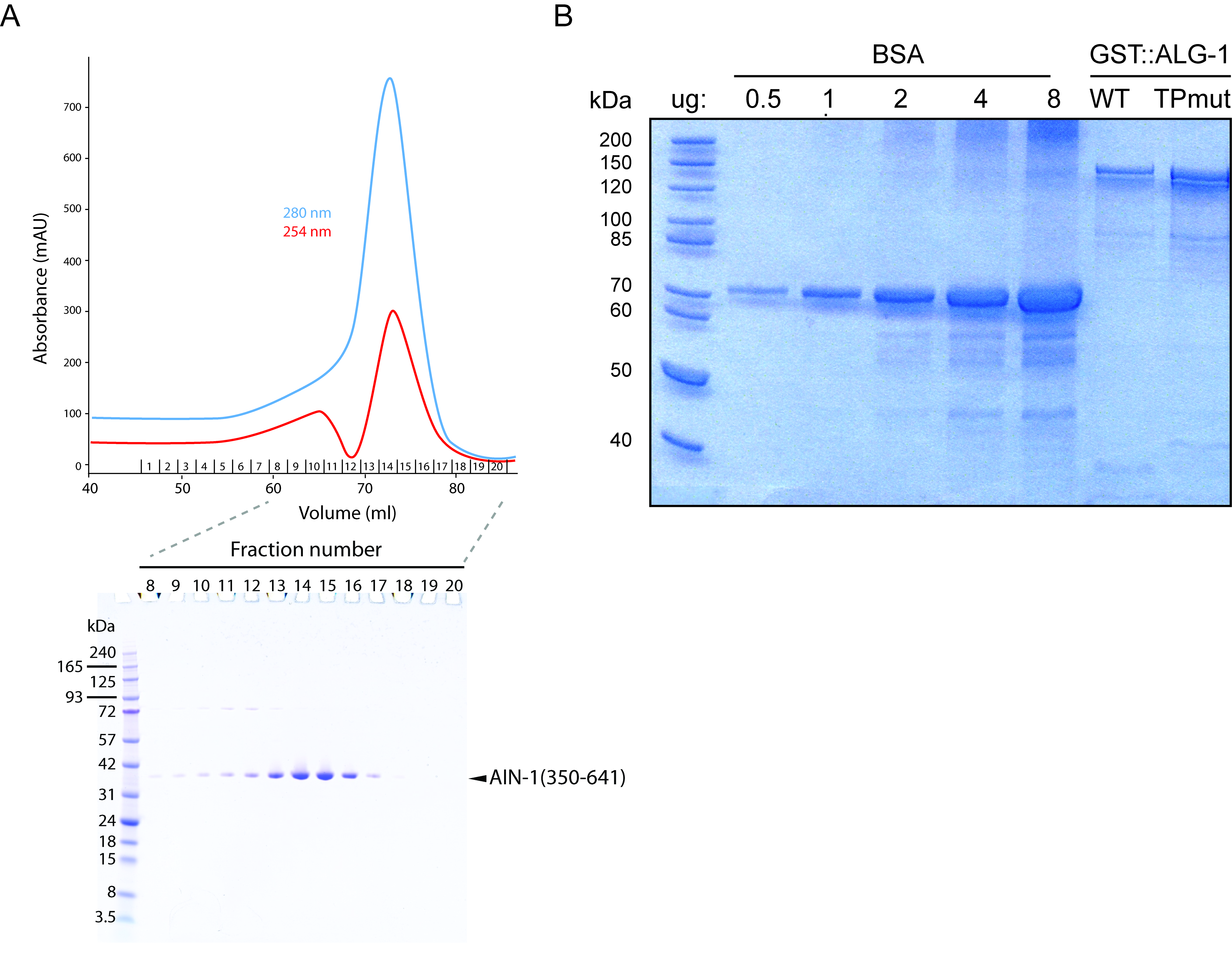

Supplement: S2 Fig — (A) Analyses of AIN-1 (350–641) using size exclusion chromatography (Top) and the SDS-PAGE (Bottom). (B) Complementary DNA (cDNA) of alg-1 was fused to glutathione S-transferase (GST) into a pGEX expression vector and bacterially expressed after induction by IPTG. ALG-1(TPmut) was obtained by directed mutagenesis using pGEX::alg-1(wt) as template. The cytosolic extract of E. coli strain expressing GST-Tagged ALG-1(WT) or ALG-1(TPmut) was loaded onto GST-sefinose resin and analyzed by SDS-PAGE. Increasing amount of commercial recombinant Bovine Serum Albumin (BSA) was loaded in parallel. (TIF) [file pgen.1006484.s002.tif]

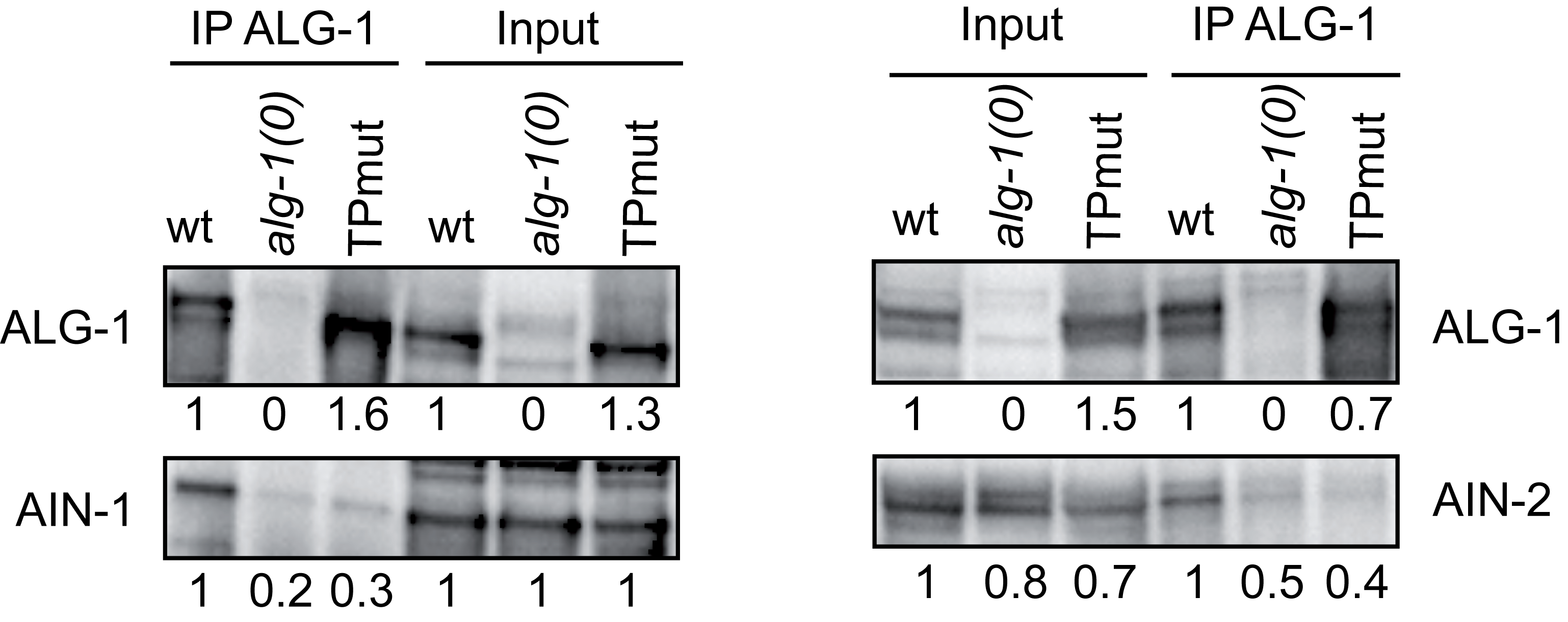

Supplement: S3 Fig — ALG-1 proteins from embryos were co-immunoprecipitated using ALG-1 specific antibody and AIN-1 and AIN-2 were detected by Western Blotting. Inputs represent the equivalent of 20% of the total protein extracts used for the IPs. The relative enrichment of ALG-1 (wt and TPmut) as well as AIN-1 and AIN-2 in the IPs normalized to respective input signals are indicated. (TIF) [file pgen.1006484.s003.tif]

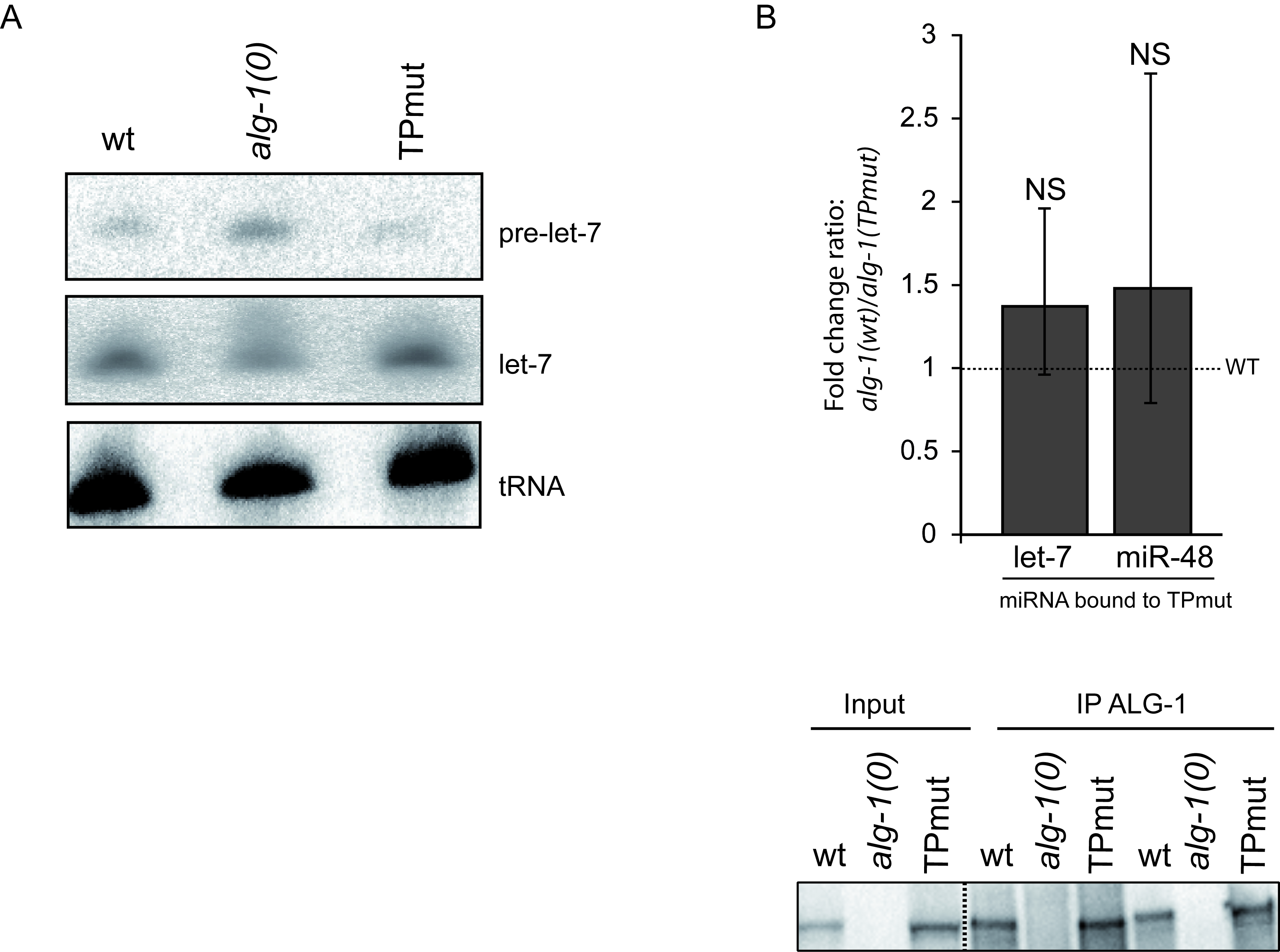

Supplement: S4 Fig — (A) Northern blot detection of let-7 miRNA in wild-type (wt), alg-1(0) and alg-1(0) animals expressing ALG-1(TPmut). Upon total RNA extraction, let-7 precursor (pre-let-7) and mature miRNA (let-7) forms were detected using probes complementary to mature miRNAs. The tRNA Glycine (tRNA) was probed and used as loading control. (B) ALG-1 immunoprecipitations from young adult animals populations expressing either ALG-1(WT) or ALG-1(TPmut) were performed using specific ALG-1 polyclonal antibody. 90% of the isolated complex was used from RNA extraction and quantitative RT-PCR analysis (Top) whereas the 10% remaining was analyzed by immunoblotting against ALG-1 (Bottom). For controls, immunoprecipitations were also performed with alg-1(0) animals. Inputs represent 5% of total protein extracts used for immunoprecipitation. The dashed line indicates that unrelated lanes have been removed between samples. RNA bounds immunoprecipitated complexes were extracted and the level of let-7 and miR-48 was quantified by quantitative RT-PCR. Before extraction, samples were spiked with human miR-20a microRNA and used as a technical control. Data was normalized to ALG-1(WT). The error bars represent the 95% confidence interval from two independent experiments and a Student’s two-sided t-test was applied on the normalized Ct values to obtained p values. NS = no significance. (TIF) [file pgen.1006484.s004.tif]

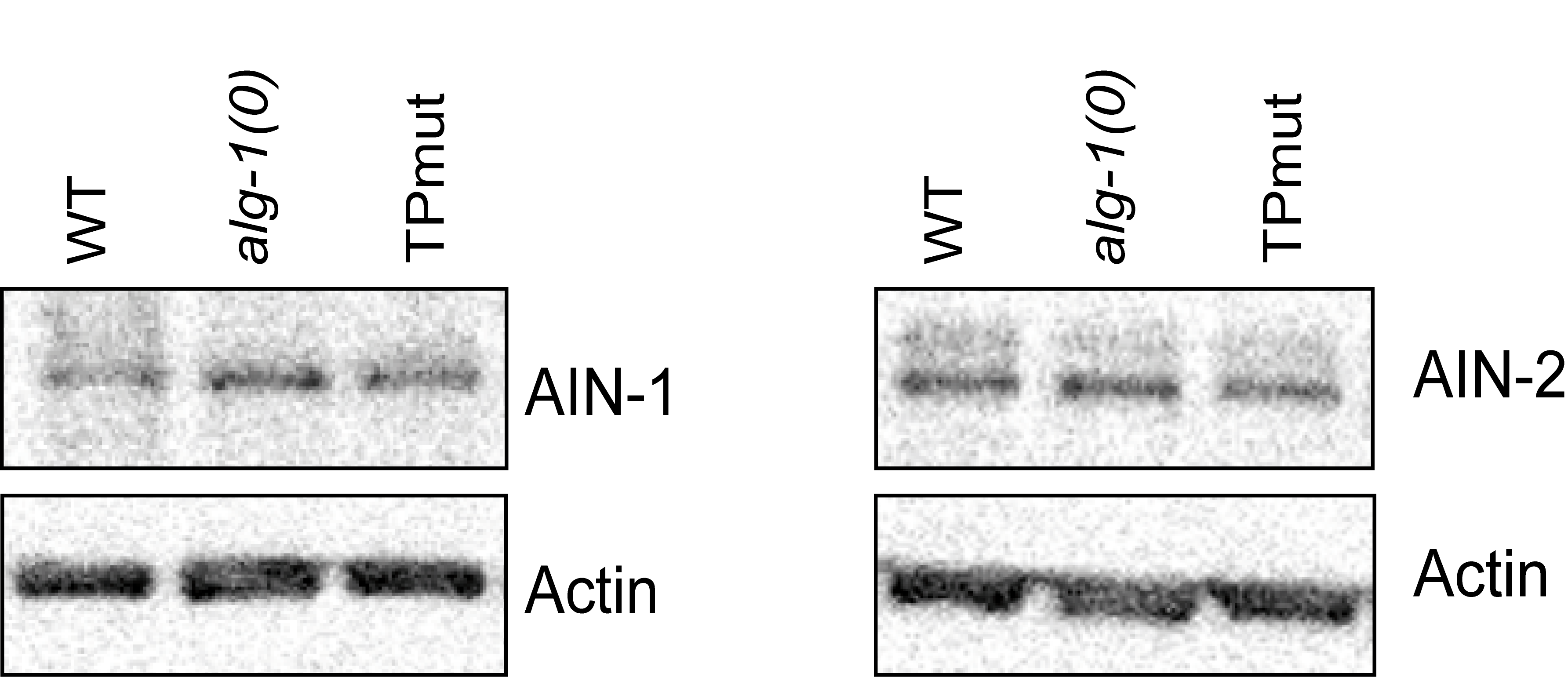

Supplement: S5 Fig — Animals population expressing either ALG-1(TPmut) or ALG-1(WT) along with alg-1 null allele alg-1(0) were boiled into SDS loading buffer and loaded onto SDS-PAGE gel for analysis of endogenous AIN-1 (left) and AIN-2 (right) protein levels. Actin was used as a loading control. (TIF) [file pgen.1006484.s005.tif]

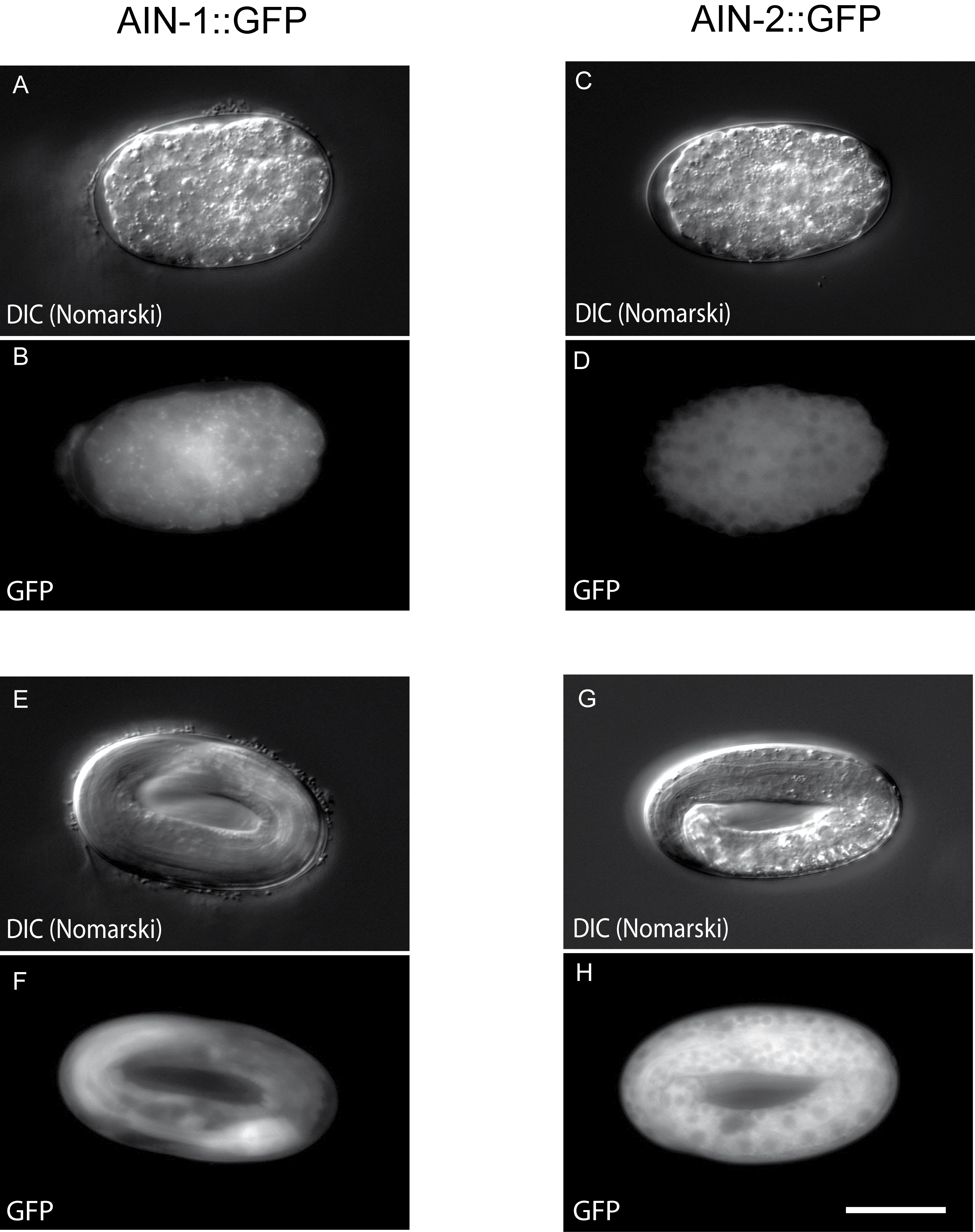

Supplement: S6 Fig — (B-D-F-H) Embryos from transgenic worms expressing GFP-tagged AIN-1 or AIN-2 were observed under fluorescent microscope at 400X magnification with 1000ms of exposure (with the exception of panel B: 2000ms) and with Nomarski optics (A-C-E-G). Scale bar: 20 μm. (TIF) [file pgen.1006484.s006.tif]
