# Supplementary material for: GW182-Free microRNA Silencing Complex Controls Post-transcriptional Gene Expression during Caenorhabditis elegans Embryogenesis
Source: PLoS Genet. 2016 Dec 9;12(12):e1006484. doi: 10.1371/journal.pgen.1006484 (PMC5147811; doi:10.1371/journal.pgen.1006484)
Supplement: S1 Table — (DOCX) [file pgen.1006484.s007.docx]

***Table S1****: List of oligonucleotides primers used in this study.*

| **Primers** | **Sens** | **Insert to amplify** | **Sequence** |
| --- | --- | --- | --- |
| MSo1268 | 5’ | *cog-1* 3’UTR 1st part | 5’- attgctgaattccttttaagcgttctacctctcc -3’ |
| MSo1269 | 3’ | *cog-1* 3’UTR 1st part | 5’- attgctgcggccgctggctataactatgggaaaac -3’ |
| MSo1270 | 5’ | *cog-1* 3’UTR 2nd part | 5’- attgctgcggccgcatttcccatttaattgtacg -3’ |
| MSo1271 | 3’ | *cog-1* 3’UTR 2nd part | 5’- attgctcttaagcaattttccagaaagccgaag -3’ |
| MSo1389 | 5’ | *alg-1* promoter | 5’- tagcgagctcggaagaaaaaccacccccttgg -3’ |
| MSo1390 | 3’ | *alg-1* promoter | 5’- attgctggatcctgggtcgtttgaggcgacgttagacg -3’ |
| MSo1274 | 5’ | Box-B | 5’- attgctgcggccgccctgttcctgtagcccggggat -3’ |
| MSo1275 | 3’ | Box-B | 5’- attgctgcggccgctacaagttgaactgtactctagaac -3’ |
| MSo1654 | 5’ | *alg-1::gfp::cog-1-box-B* | 5’- attgctaggcctgaaacccaaccatcgaccctcgac -3’ |
| MSo1653 | 3’ | *alg-1::gfp::cog-1-box-B* | 5’- attgctctgcagggtgtccggcaacataattcc -3’ |
| MSo1141 | 5’ | mCherry | 5’- atagcggccgcgatggtctcaaaggg -3’ |
| MSo1142 | 3’ | mCherry | 5’- atggcggccgccttatacaattcatcc -3’ |
| MSo1878 | 5’ | P733A | 5’- gcccaagaatcttcaacgaagcagtcattttctttggatg -3’ |
| MSo1879 | 5’ | F802A | 5’- ctaccgcaacactcgcgccaagccagccagaatc -3’ |
| MSo1880 | 5’ | K803A | 5’- ccgcaacactcgcttcgcgccagccagaatcgtt -3’ |
| MSo1881 | 5’ | E838A | 5’- ggcttgcatgatgcttgcaagaggatatcaaccag -3’ |
| MSo1892 | 5’ | F802A;K803A | 5’- caattctaccgcaacactcgcgccgcgccagccagaatcgttgtcta -3’ |
| MSo1386 | 5’ | *λN-mcherry* | 5’- tagcgcggccgccgatgcgcagac -3’ |
| MSo1387 | 3’ | *λN-mcherry* | 5’- tagcgcggccgccttatacaattcatccatgc -3’ |
| MSo2204 | 5’ | *ain-2* | 5’- tggcggccgctctagaatgaacggggacggttgg -3’ |
| MSo2205 | 3’ | *ain-2* | 5’- tatagggcgaattgggtacccatcagcatcaccccatcc -3’ |
| MSo0384 | 5’ | *alg-2* | 5’- attgctaatacgactcactatagggtctagaattcgctgacatcgtcgtcttt -3’ |
| MSo0395 | 3’ | *alg-2* | 5’- attgctaatacgactcactatagggggtaccgagaaggaggagatcaggca -3’ |
| ssDNA1 | 5’ | ssDNA Box-B | 5’- gatctgggccctgaagaagggcccggatccgaactagtgggccctgaagaagggccct -3’ |
| ssDNA2 | 3’ | ssDNA Box-B | 5’- ctagagggcccttcttcagggcccactagttcggatccgggcccttcttcagggccca -3’ |
| ssDNA3 | 5’ | ssDNA λN | 5’- ggccagcgatgcgcagacccgccgccgcgaacgccgcgcggaaaaacaggcgcagtggaaagcggcgaacgc -3’ |
| ssDNA4 | 3’ | ssDNA λN | 5’- ggccgcgttcgccgctttccactgcgcctgtttttccgcgcggcgttcgcggcggcgggtctgcgcatcgct -3’ |
