# Supplementary material for: GW182-Free microRNA Silencing Complex Controls Post-transcriptional Gene Expression during Caenorhabditis elegans Embryogenesis
Source: PLoS Genet. 2016 Dec 9;12(12):e1006484. doi: 10.1371/journal.pgen.1006484 (PMC5147811; doi:10.1371/journal.pgen.1006484)
Supplement: S2 Table — (DOCX) [file pgen.1006484.s008.docx]

***Table S2****: List of plasmids used in this study.*

| **Plasmid** | **Backbone** | **Insert** |
| --- | --- | --- |
| MSp0343 | pPD95­_75 | *alg-1p::gfp::cog-1-box-B* |
| MSp0094 | pCFJ178 | *alg-1p::gfp::cog-1-box-B* |
| MSp0186 | pBS SK+ | *alg-1p: λN::mcherry::alg-1(WT)::alg-1 3’UTR* |
| MSp0344 | pBS SK+ | *alg-1p: λN::mcherry::alg-1(TPmut)::alg-1 3’UTR* |
| MSp059 | pBS SK+ | *alg-1p:RFP::alg-1::alg-1 3’UTR* |
| MSp0164 | pCFJ151 | *alg-1p::mcherry::alg-1(WT)::alg-1 3’UTR* |
| MSp0347 | pCFJ151 | *alg-1p::mcherry::alg-1(TPmut)::alg-1 3’UTR* |
| MSp0348 | pCFJ151 | *alg-1p:: λN :mcherry::alg-1(WT)::alg-1 3’UTR* |
| MSp0349 | pCFJ151 | *alg-1p:: λN :mcherry::alg-1(TPmut)::alg-1 3’UTR* |
| MSp0248 | pGEX 6P-1 | *alg-1* cDNA |
| MSp0350 | pGEX 6P-1 | *alg-1(TPmut)* cDNA |
| MSp0266 | L4440 | *alg-2* *(N-ter)* |
| MSp0343 | L4440 | *ain-2* |
