# Supplementary material for: GW182-Free microRNA Silencing Complex Controls Post-transcriptional Gene Expression during Caenorhabditis elegans Embryogenesis
Source: PLoS Genet. 2016 Dec 9;12(12):e1006484. doi: 10.1371/journal.pgen.1006484 (PMC5147811; doi:10.1371/journal.pgen.1006484)
Supplement: S1 Text — (DOCX) [file pgen.1006484.s009.docx]

**GW182-free microRNA silencing complex controls post-transcriptional gene expression during *C .elegans* embryogenesis**

Guillaume Jannot, Pascale Michaud, Miguel Quévillon Huberdeau, Louis Morel-Berryman, James A. Brackbill, Sandra Piquet, Katherine McJunkin, Kotaro Nakanishi and Martin J. Simard

SUPPLEMENTAL INFORMATION

SUPPLEMENTAL MATERIALS AND METHODS

***C. elegans* strains generated for this study**

| **Strain** | **Genotype** |
| --- | --- |
| **MJSSi03** | *qbcSi03[alg-1p::gfp::cog-1-boxb;cb-unc-119(+)]IV* |
| **MJSSi04** | *qbcSi04[alg-1p::mcherry::alg-1(tpmut)::alg-1;cb-unc-119(+)]V* |
| **MJSSi05** | *qbcSi05[alg-1p::λN::mcherry::alg-1::alg-1;cb-unc-119(+)]V* |
| **MJSSi06** | *qbcSi06[alg-1p::λN::mcherry::alg-1(TPmut)::alg-1;cb-unc-119(+)]V* |
| **MJSEx01** | *alg-1(gk214) qbcEx01[alg-1p:: λN::mcherry::alg-1(TPmut)::alg-1 3’UTR; pRF4(rol-6(su1006))]* |
| **MJSEx04** | *alg-1(gk214) qbcEx04[alg-1p:: λN::mcherry::alg-1:alg-1 3’UTR; pRF4(rol-6(su1006))]* |
| **MJS041** | *qbcSi03[alg-1p::gfp::cog-1-boxb;cb-unc-119(+)]IV ; qbcSi05[alg-1p::λN::mcherry::alg-1::alg-1 3’UTR;cb-unc-119(+)]V* |
| **MJS117** | *qbcSi03[alg-1p::gfp::cog-1-boxb;cb-unc-119(+)]IV ; qbcSi06[alg-1p::λN::mcherry::alg-1(TPmut)::alg-1 3’UTR; cb-unc-119(+)]V* |
| **MJS019** | *alg-1(gk214) qbcSi05[alg-1p::λN::mcherry::alg-1::alg-1 3’UTR;cb-unc-119(+)]V* |
| **MJS014** | *alg-1(gk214) qbcSi04[alg-1p::mcherry::alg-1(TPmut)::alg-1 3’UTR;cb-unc-119(+)]V* |
| **MJS118** | *alg-1(gk214) qbcSi06[alg-1p::λN::mCherry::alg-1(TPmut)::alg-1 3’UTR;cb-unc-119(+)]V* |
| **MJS119** | *alg-1(gk214) mjsSi04[alg-1p::mcherry::alg-1(TPmut)::alg-1 3’UTR;cb-unc-119(+)]V; ctIs39[hbl-1::GFP; pRF4(rol-6(su1006))]* |
| **MJS142** | *ain-1(ku322); ain-2(tm2432)/dpy-5(e61)* |
| **MJS173** | *alg-1(gk214) In[col-10p::gfp::lin-41 3'UTR]* |
| **MJS174** | *alg-1(gk214) qbcSi06[alg-1p::λN::mCherry::alg-1(TPmut)::alg-1 3’UTR;cb-unc-119(+)]V ) In[col-10p::gfp::lin-41 3'UTR]* |
| **MJS189** | *her-1(n695);alg-1(gk214) qbcSi06[alg-1p::λN::mCherry::alg-1(TPmut)::alg-1 3’UTR;cb-unc-119(+)]V)* |
| **MJS190** | *her-1(n695);alg-1(gk214)* |

***Molecular cloning of transgenes****Construction of GFP:: Box-B reporter vectors*

Complementary single stranded DNA containing 2 copies of Box-B sequence was annealed and cloned three times successively in pBS SK+ vector using BamHI and SpeI restriction sites to form a fragment containing 6 copies of Box-B. pPD95_75 vectors (kindly provided by Dr Hobert) was digested with EcoRI and AflII restriction enzymes and ligated with a fragment obtained by ligation of two PCR products of *cog-1* 3’UTR (primers colored in bold) where the *lsy-6* miRNA binding (colored in red) portion was deleted (represented in italic) and replaced by a NotI restriction site. A 6 copies of Box-B PCR amplified fragment flanked with NotI restriction site was cloned into the NotI site. The *ceh-36* promoter was replaced by a 3200bp of PCR amplified *alg-1* promoter flanked with SacI and BamHI restriction sites to produce MSp0343. The final construction was PCR amplified and cloned into pCFJ178 vector with PstI/StuI restriction sites to produce MSp0094.

Sequence of *cog-1* 3’UTR

5’-**cttttaagcgttctacctctcc**ccctcccttcaaccgagtgtattattcccccaatttgtttgcaattttttcctgaagccctttaagaaaatccaaaatcatgaccttcttccgtctttacacctgattacctgaataccaacaccccacacagatgccatgatctctcgtcttttctcgtacttttgtataatttttttcttaatttttttgcat**gttttcccatagttatagcca***tttttttttctttttttttccaaatcatcgtcacttatacaaaaaccaaactcccttttaccgttaaaccatgcccaaatacaaaa***aatttcccatttaattgtacg**tttttttctcttcaaattggattctaatgacataaatttattagattaagtaatcgttgaggcatatttacagataccaaatattgtggggaacacgaaaaattaattaattatctattcgagtttcgttttgatcgtctggagaacgttcatgagcacgtcgagggtcgatggttgggttt**cttcggctttctggaaaattg**

-3’

*Construction of ALG-1 expression vectors used as Extrachromosomal arrays for rescue experiments.*

The pBS SK+ GFP::ALG-1 vector was digested with NotI restriction enzyme to remove GFP and ligated with a PCR amplified mCherry tag from pCFJ104 where the 5’ NotI site was abolished. The λN coding sequence was then inserted using the remaining NotI site to obtain MSp0186. The tryptophan binding mutations (MSp0344) were generated using The QuikChange Multi Site-Directed Mutagenesis Kit (see Table S1 for primers).

*Construction of mosSCI targeting vectors*

A digested AflII/Acc65I fragment from MSp059 plasmid containing N-terminal RFP tagged *alg-1* coding sequence flanked with 3941pb and 2958pb *alg-1* 5’ and 3’ UTRs respectively was ligated into pCFJ151 digested with AflII/BsiWI restriction enzymes. Using NotI restriction sites, the RFP tag was removed and replaced with PCR amplified mCherry from pCFJ104 to produce MSp164 or λN-mCherry from MSp186 to produce MSp0348. The tryptophan binding mutations in MSp0347 and MSp0349 were generated using The QuickChange Multi Site-Directed Mutagenesis Kit (see Table S2).

*Construction of GST-tag ALG-1 expression vectors*

The tryptophan binding mutations were generated using The QuikChange Multi Site-Directed Mutagenesis Kit using ALG-1 cDNA cloned into pGEX 6P-1 as template [22].
